# Yellow fever in Pará State, Amazon region of Brazil, 1998-1999: entomologic and epidemiologic findings.

**DOI:** 10.3201/eid0707.010738

**Published:** 2001

**Authors:** P F Vasconcelos, A P Rosa, S G Rodrigues, E S Rosa, H A Monteiro, A C Cruz, V L Barros, M R Souza, J F Rosa

**Affiliations:** Instituto Evandro Chagas, FUNASA, Ministry of Health of Brazil, Belém, PA, Brazil. pedrovasconcelos@iec.pa.gov.br

## Abstract

Yellow fever (YF) is frequently associated with high severity and death rates in the Amazon region of Brazil. During the rainy seasons of 1998 and 1999, 23 (eight deaths) and 34 (eight deaths) human cases of YF were reported, respectively, in different geographic areas of Pará State; most cases were on Marajó Island. Patients were 1 to 46 years of age. Epidemiologic and ecological studies were conducted in Afuá and Breves on Marajó Island; captured insects yielded isolates of 4 and 11 YF strains, respectively, from Haemagogus janthinomys pooled mosquitoes. The cases on Marajó Island in 1999 resulted from lack of vaccination near the focus of the disease and intense migration, which brought many nonimmune people to areas where infected vectors were present. We hypothesize that YF virus remains in an area after an outbreak by vertical transmission among Haemagogus mosquitoes.


### Yellow Fever in Pará State, Amazon Region of Brazil, 1998–1999: Entomologic and Epidemiologic Findings

**Pedro F. C. Vasconcelos,* Amélia P.A.T. Rosa,*† Sueli G. Rodrigues,* Elizabeth S.T. Rosa,* Hamilton A.O. Monteiro,* Ana C.R. Cruz,* Vera L.R.S. Barros,* Maria R. Souza,* and Jorge F.S.T. Rosa***
*Instituto Evandro Chagas, FUNASA, Ministry of Health of Brazil, Belém, PA, Brazil; and †University of Texas Medical Branch, Galveston, Texas, USA

Yellow fever (YF) is frequently associated with high severity and death rates in the Amazon region of Brazil. During the rainy seasons of 1998 and 1999, 23 (eight deaths) and 34 (eight deaths) human cases of YF were reported, respectively, in different geographic areas of Pará State; most cases were on Marajó Island. Patients were 1 to 46 years of age. Epidemiologic and ecological studies were conducted in Afuá and Breves on Marajó Island; captured insects yielded isolates of 4 and 11 YF strains, respectively, from *Haemagogus janthinomys* pooled mosquitoes. The cases on Marajó Island in 1999 resulted from lack of vaccination near the focus of the disease and intense migration, which brought many nonimmune people to areas where infected vectors were present. We hypothesize that YF virus remains in an area after an outbreak by vertical transmission among *Haemagogus* mosquitoes.

Yellow fever (YF) is an important arbovirus infection of humans and sylvan primates. Infection in humans is accompanied by high rates of illness and death. The causative agent, YF virus, is the prototype strain of the genus *Flavivirus*, family Flaviviridae (1). YF virus is maintained in two distinct transmission cycles. One is sylvatic: monkeys act as vertebrate hosts, forest canopy mosquitoes of the genera *Haemagogus* and *Sabethes* (chiefly *Haemagogus janthinomys* and to a lesser extent *Sabethes chloropterus)* are vectors, and human infections occur as sporadic cases or as limited outbreaks (2-4). The other transmission cycle is urban: YF virus is directly transmitted from human to human by the bite of infected *Aedes aegypti* mosquitoes, and other animals are not associated with transmission. Urban YF was eradicated from Brazil after 1942 (3-5). Sylvan YF virus is still reported in South America and Africa (6-9).

In recent decades in South America, YF cases and outbreaks have been reported, especially in Peru, Bolivia, and Brazil, where >90% of all reported episodes of disease in the 1990s occurred (10). In Brazil, YF occurs annually, causing sporadic cases, small outbreaks, or self-limited epidemics in the jungle or rural areas of the Amazon region (the natural focus of the disease), the Central-West region, and western regions of Maranhão and Minas Gerais states (3,4,11).

We report entomologic and epidemiologic findings regarding an unusual occurrence of YF cases in an area of Pará State in 1998 and 1999.

Address for correspondence: Pedro Fernando da Costa Vasconcelos, Instituto Evandro Chagas, Av. Almirante Barroso, 492, 66090-000, Belém, PA, Brazil; fax: 5591-226-1284/226-5262; e-mail: pedrovasconcelos@iec.pa.gov.br

### Materials and Methods

### Collection Sites

Mosquito and blood collections were made at three sites in Pará State (Figure 1). Afuá (0°06' S; 50°20' W; population approximately 30,000) and Breves (1°41' S, 50°19' W); population approximately 75,000, are municipalities of Marajó Island, in the northern region of Pará State, known for buffalo breeding and fish farming. The other site, Altamira (2° 51' S; 51° 57' W) (approximately 80,000 inhabitants), is in the central region of the state on the Xingu River delta near the Transamazon Highway; its chief products are wood, cattle, and sugar cane and cacao.

### Samples

Blood samples were taken from persons who had clinical symptoms and signs compatible with YF for attempts at virus isolation, as well as from contacts (family and neighbors) for serologic examinations. Approximately 5 mL to 10 mL of blood was obtained by venipuncture. Serum samples were stored at -20°C until tested. From patients with fever and other clinical symptoms, specimens were also obtained for attempted virus isolation. Monkeys were euthanized, and blood samples and liver fragments obtained for virus isolation. All specimens were frozen in liquid nitrogen containers until processed in the laboratory.

### Mosquitoes

Diurnal human-biting mosquitoes were collected in Afuá, Breves, and Altamira. The collections were made from 9:00 a.m. to 3:00 p.m. on the ground and at an elevation of approximately 15 m in the forest canopy, since these are the more active periods and places of potential YF virus vectors.

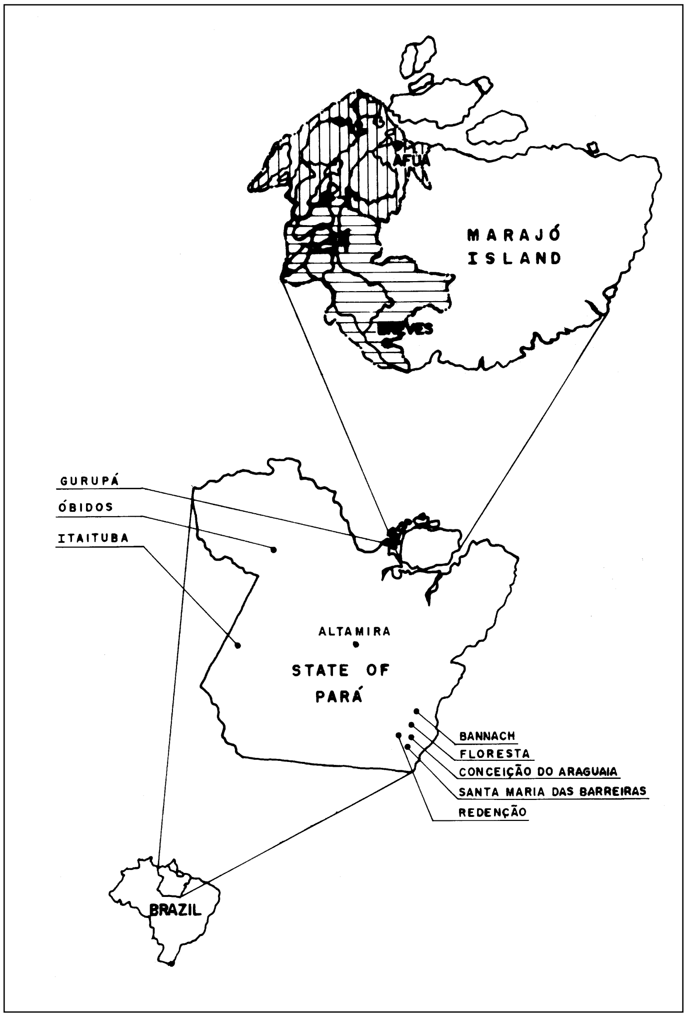

Figure 1. Map of Pará State showing Afuá, Breves and other municipalities where yellow fever was reported in 1998 and 1999, and Altamira municipality where yellow fever virus was isolated in mosquitoes and monkeys.

In Afuá, collections were made from May 17 to May 30, 1998, and from March 11 to March 25, 1999. Collections in Breves were from March 11 to March 25, 1999. In Altamira, collections were made on April 16 to April 28, May 20 to June 5, and July 10 to July 19, 1998, and from January 31 and February 13 (rainy season) and July 14 to July 27 (dry season) in 1999.

In the laboratory, mosquitoes were classified and pooled under refrigeration, by species, place, and dates of collection, and then preserved at -70°C until inoculation. Minimum infection rates (MIRs) of *Hg. janthinomys* mosquitoes were calculated by dividing the total of positive pools by the total number of specimens processed (12).

### Serology

Serum samples were initially screened by hemagglutination inhibition (HI) against YF antigen (strain Be H 111). Tests were performed as described by Clarke and Casals, using a microtechnique in which serum samples were acetone extracted (13). All positive samples were later assayed by enzyme immunoassay for capture of immunoglobulin (Ig) M (MAC-ELISA) (14). All positive samples by both tests were later tested by plaque reduction neutralization test (PRNT) to confirm infection (15).

### Serologic Criteria for Inclusion in Study

Positive diagnostic criteria for inclusion in our study were 1) serologic conversion by fourfold increase in antibody titers between acute- and convalescent-phase (7 to 14 days after first collection) serum samples and 2) presence of IgM without history of YF vaccination plus positive reaction (>1:10) by PRNT.

### Pathology

From fatal cases, liver samples were obtained; histologic sections were stained by hematoxylin and eosin and examined by light microscopy. Specific YF antigens were detected in paraffin-embedded liver samples of fatal cases by means of an immunohistochemistry technique (16). All patients with these antigens were considered YF-positive cases and included in the study.

### Virus Isolation and Identification

All samples (blood, viscera, and mosquitoes) were inoculated into suckling mice and C6/36 cells for virus isolation. Before processing, samples were thawed, triturated, and diluted in phosphate-buffered saline (pH 7.4) with 0.75% of bovine albumin and antibiotics (100 µg/mL of streptomycin and 100 IU/mL of penicillin) and centrifuged for 10 minutes at 2,100 x *g* (15). The supernatant of each specimen was then injected into suckling mice (0.02 mL intracerebrally) and into tubes of cells (0.1 mL), respectively. Isolated strains were identified by indirect immunofluorescence assay and complement fixation test (15). Isolation of YF virus from blood or tissues of human patients without history of vaccination was used as the positive criterion for inclusion in the study.

### Results

### Epidemiology

In 1998 and 1999 in Pará State, 23 and 34 YF cases, respectively, were diagnosed. In 1998, 17 of the 23 occurred in Afuá and 6 in municipalities not on Marajó Island (Bannach, Floresta, Gurupá, Itaituba, Óbidos, and Redenção). In 1999, 15 of the 34 diagnosed cases occurred in Afuá, 14 in Breves, and 5 in three other municipalities (Conceição do Araguaia, 2; Santa Maria das Barreiras, 2; Redenção, 1), all in the southeast region (Figure 1). The sex and age distributions of cases were determined (Figure 2). Comparison of total cases and deaths in both years shows a case-fatality rate of 34.8% in 1998 and 23.5% in 1999 (Figure 3). Most reported cases were diagnosed by serology or another technique combined with serology, with clinical and epidemiologic features taken into account (Figure 4).

#### Afuá

A scientific trip was undertaken to the municipality (May 17 to 25, 1998). From the 23 human samples, three isolations of the YF virus (H 603325, H 603327, and H 603797) were made. Of the monkey specimens, two samples of YF virus (AN 604552, AN 604555), were isolated from the blood and liver, respectively of monkey (PR 2968) of the species *Alouatta belzebul*. The YF cases occurred in several areas, among them

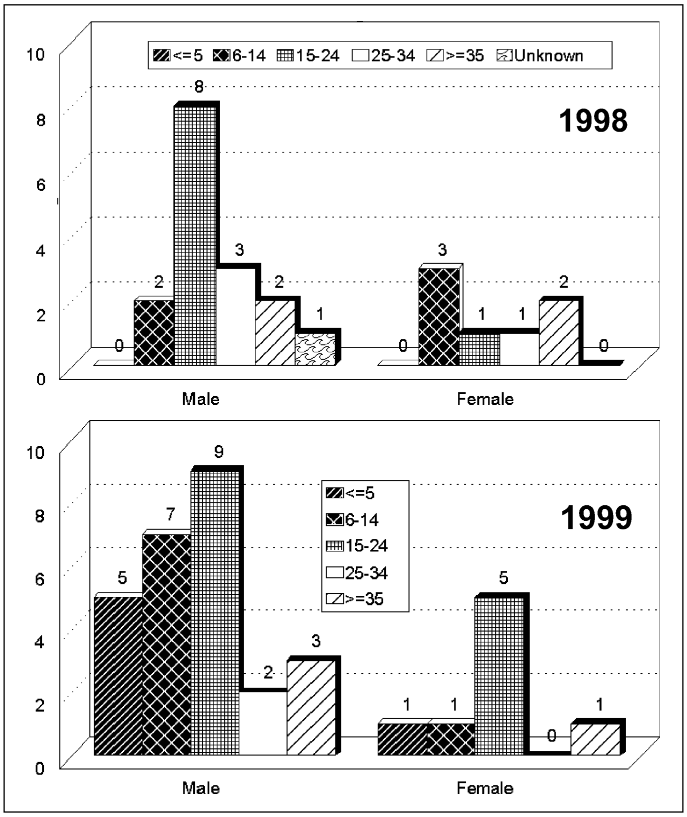

Figure 2. Distribution of yellow fever cases, Pará State, by age groups, 1998 and 1999.

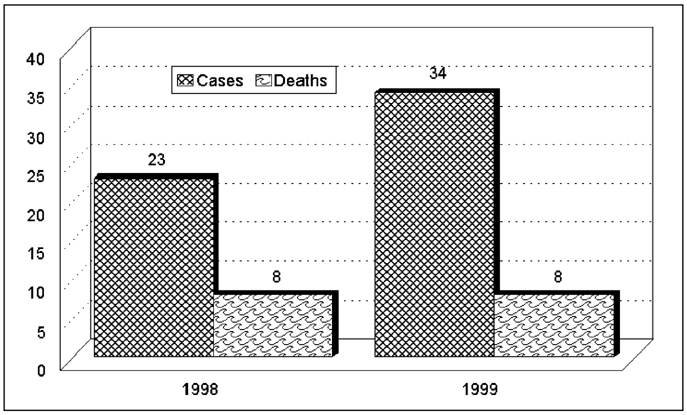

Figure 3. Yellow fever cases and deaths reported, Pará State, 1998–1999.

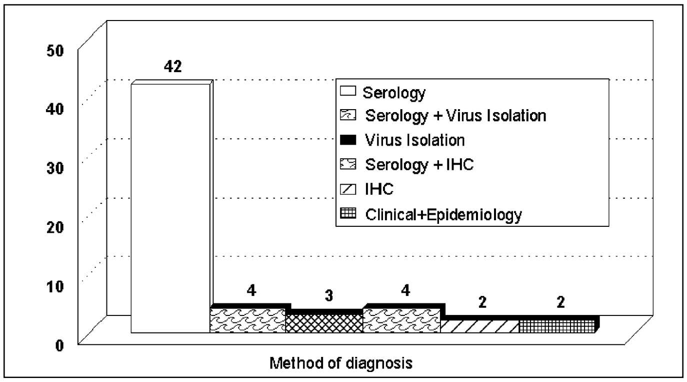

Figure 4. Diagnostic procedures used on the yellow fever cases reported, Pará State, 1998–1999. IHC = immunohistochemistry.

the river Morcego, Morceguinho, Tamanduá, Bom Jardim, and Furo da Cidade.

### Entomology

### Afuá

A total of 1,621 Culicidae were collected with human bait during the day in the canopy and at ground level, which after identification, provided 77 pools for inoculation. The most abundant species were *Wyeomyia* sp and *Hg. janthinomys,* with 1,119 (69%) and 296 (18.3%) specimens, respectively; after identification, these formed 23 and 14 pools, respectively, for virus isolation in tissues. Four samples of YF virus were isolated from pools of *Hg. janthinomys* (AR 605158, AR 605159, AR 605160, and AR 605161). The MIR for *Hg. janthinomys* was 1.35%.

### Altamira

Deaths of monkeys in the forest on agricultural secondary roads of the Transamazon Highway in the stretch from Altamira to Marabá (km 20 and 27) motivated a scientific expedition from April 16 to 28, 1998. A total of 592 hematophagous insects were collected on human bait; 479 (80.9%) were the mosquito *Hg. janthinomys*, which after identification provided 38 and 24 lots, respectively, for virus research. The pools inoculated yielded 10 isolations of YF virus, all from *Hg. janthinomys* (MIR = 2.01%).

The high infection rate with YF virus observed in *Hg. janthinomys* motivated a second trip (May 20-June 5) to determine the spatial distribution and infection rate of *Hg. janthinomys*, as well as to evaluate the dynamics of the circulation of YF virus in the Altamira area. A total of 509 hematophagous diptera (66 lots) were captured; 312 (61.3%) (29 lots) belonged to the species *Hg. janthinomys*. Three samples were identified as YF virus, as occurred in the first trip; all positive samples were from pools of *Hg. janthinomys*, for an MIR of 0.96%.

From July 10 to July 19 (dry season), a third trip was made to the same area of Altamira to monitor circulation of YF virus. In this trip, 120 hematophagous insects were captured in 14 lots; 28 (23.3%) of them were *Hg. janthinomys* from a single lot. Injection of these lots into newborn mice did not produce virus.

In the rainy season (January 31 to February 13) of 1999, 1,105 (93 pools) mosquitoes were captured; 84 (5 pools) were *Hg. janthinomys*. During the dry season (July 14 to 27), 133 mosquitoes (14 pools) were collected; 44 (3 pools) were *Hg. janthinomys*. No virus was isolated.

### Afuá/Breves

The occurrence of human cases motivated the expedition to Afuá and Breves to carry out entomologic studies of potential vectors of YF virus. From March 11 to March 25, 1999, captures of hematophagous insects were performed at ground level and in the forest canopy. A total of 2,164 insects were collected on human bait in 126 pools; 546 of these were *Hg. janthinomys*, which furnished 23 pools for inoculation in attempts at virus isolation. Eleven strains of YF virus were obtained from pools of *Hg. janthinomys*, for an MIR of 2.01%. No virus was isolated from the other mosquito pools. During this trip, three howler monkeys (*Alouatta belzebul*) were euthanized. The specimens from the monkeys produced three

YF virus isolates, two from the blood and liver of the same monkey and another from the liver of a second monkey. These monkeys were found within 200 m to 500 m of human dwellings. They had been showing abnormal behavior, i.e., moving slowly and not trying to escape from people.

### Discussion

In 1998, in Afuá municipality, the first YF human case (based on epidemiologic information) had onset of symptoms on February 3. As the YF medium incubation period ranges from 3 to 6 days, infection probably occurred at the end of January. Our trip to the municipality was in May, >3 months after the index case. Despite the long interval between the index case and our expedition, we recovered YF virus from pools of *Hg. janthinomys* mosquitoes and from howler monkeys. These findings strongly suggest an elevated natural average infection rate of the vector mosquitoes in the area. This was the first detection of YF virus in the municipality of Afuá. YF virus has not been reported on Marajó Island since 1988, when a sporadic case occurred near Breves in a man who cut down a tree.

The study in Altamira in 1998 shows clearly how YF virus outbreaks happen. The rainy season, in the first months of the year, has the highest rainfall indexes in the Amazon forest region. This facilitates breeding of mosquitoes, including the potential vector *Hg. janthinomys*, in the forest. When the rains decrease, the amount of mosquitoes in the forest gradually decreases (Table). As the population of mosquito vectors decreases, YF virus disappears.

In Breves and Afuá in 1999, reported cases clearly resulted from a failure of the vaccination campaign, since after the outbreak in 1998, the inhabitants of Afuá and Breves were vaccinated. However, some people from Afuá were not immunized, and they migrated to areas near places where cases had been previously reported. The occurrence of these cases and the isolation of YF virus from *Hg. janthinomys* and monkeys indicate intense circulation of the virus. Circulation of the virus for 2 years in the same limited area probably occurred because eggs of infected mosquitoes remained in the region. When the rainy season began, the *Hg. janthinomys* eggs hatched, and probably many of them were born infected by vertical transmission. Although little evidence of this possibility has been obtained in nature, it is plausible because the monkey population is not thought to be large enough to maintain the sylvan cycle (17). Effectively, monkeys who are acutely infected either die or become immune, aborting further infections (8,18). On the other hand, evidence for smaller mammals (especially rodents and marsupials) playing a role in the maintenance cycle has been only rarely established (18-20). In Afuá and Breves municipalities, no evidence was found to incriminate hosts other than primates in the epidemic.

The current theory for YF is that transmission occurs in cyclic waves of 7 to 10 years that result in epidemics (8,18). Our results, especially in Altamira, do not confirm that observation. Based on our results, we speculate that the occurrence of epidemics in the same limited geographic region of two neighboring municipalities was only possible because mosquitoes were born infected by vertical transmission. Infections in monkeys in 1998 should have made a large number of them immune, and the short interval between the outbreaks was not enough to renew the monkey population. We hypothesize that the persistence of YF virus in a region occurs by passing through several generations of mosquitoes and that this is the main mechanism responsible for maintenance of the virus and not the epidemic wave as has been suggested (11,18-20). The occurrence of YF cases or outbreaks, therefore, is a direct function of migration of nonimmune persons. In places such as Altamira, where people show high YF vaccination rates, it is quite difficult to find a human case, despite the municipality's situation in the endemic area with virus circulation.

The absence of human YF, however, is not enough to avert virus circulation because humans acquire infection accidentally and are a deadend host, playing an unimportant role in the maintenance of the virus in nature. Continued entomologic and epidemiologic studies must be conducted in different sites where YF outbreaks or cases have been reported to prove that *Haemagogus* mosquitoes maintain YF virus vertically.

The occurrence of YF cases in other areas at the same time or within a short time period also supports our hypothesis. Since some municipalities are located >1,500 km from Afuá and Breves municipalities (Figure 1), YF virus must be present there; when nonimmune people enter the forest, they become infected. Therefore, appearance of cases is the result of a silent restricted circulation of the virus in an area's forest.

On the other hand, YF cases in South America have thus far only been transmitted by sylvatic vectors, especially *Hg. janthinomys* (3,11,21). The susceptibility of the *Ae. aegypti* population in South America to YF virus must be established, in the face of the increased risk of reemergence of urban transmission (22-24). The annual occurrence of several cases in Brazil and hundreds of them in Peru and Bolivia may have permitted contact of YF virus with *Ae. aegypti*. Surprisingly, urban transmission has not yet been reported, except for six cases in Santa Cruz, Bolivia (25). Thus, it is necessary to establish the level of infectivity and susceptibility of South American *Ae. aegypti* with YF virus.

The most important step in the control of YF would be a continent-wide resolution to improve YF vaccination (6,26,27). Protection acquired after 17D vaccine lasts up to 10 years; after that period, the Pan American Health Organization (PAHO) and World Health Organization (WHO) recommend a new dose, although some studies show protection for 17 to 35 years with a single dose (28,29). Another important measure would be creation of a network (probably Internet based) to keep all countries in the continent quickly informed of YF cases or outbreaks, especially in major risk areas.

Table. Comparison of number of mosquitoes collected on human bait, number of YF strains isolated by place of capture, and minimum infection rate (MIR) for *Haemagogus janthinomys*, Pará State, Brazil, 1998–1999

| Place | Year | Strains isolated | *Hg. janthinomys* baited | MIR | Season |
|---|---|---|---|---|---|
| Afuá | 1998 | 4 | 296 (14) | 1.35% | Rainfall |
| Altamira | 1998 | 13 | 819 (54) | 0.96%-2.01% | Rainfall |
| Altamira | 1999 | - | 129 ( 8) | - | Dry |
| Breves | 1999 | 11 | 546 (23) | 2.01% | Rainfall |

( )= No. of lots of pooled mosquitoes in which virus isolation was attempted.

To date, financing YF vaccine has been a major problem for disease-endemic countries, but studies developed in Africa have suggested that low costs are possible (26). Moreover, Brazil has been producing YF vaccine with the 17D strain on a large scale for a long time. We believe that combined efforts under PAHO/WHO support to supply other countries in the region with vaccine for a massive vaccination campaign would save thousands of lives.

### Acknowledgments

We thank the National Health Foundation (FUNASA) in Pará and Amapá States for logistic support in Afuá and Breves municipalities and NUPE (Pará State Epidemiology Nucleus) of SESPA (Department of Public Health of Pará State).

This work was financed by FUNASA (National Health Foundation) and Instituto Evandro Chagas, Ministry of Health of Brazil.

Dr. Vasconcelos works in virology, especially arboviruses, hantaviruses, and emerging and reemerging viruses. He is chief of the arbovirus section of the Instituto Evandro Chagas, FUNASA, Ministry of Health, which is a World Health Organization/Pan American Health Organization Collaborating Center for Arbovirus Reference and Research.

### References

1. Westaway EG, Briton MA, Gaidamovich SY, Horzinek MC, Igarashi A, Kaariainen L, et al. Flaviviridae. Intervirology 1985:24:183-92.
2. Pinheiro FP, Travassos da Rosa APA, Moraes MAP, Neto JCA, Camargo S, Filgueiras FP. An epidemic of yellow fever in central Brazil, 1972-1973. I. Epidemiological studies. Am J Trop Med Hyg 1978:27:125-32.
3. Dégallier N, Travassos da Rosa APA, Vasconcelos PFC, Travassos da Rosa ES, Rodrigues SG, Sá Filho GC, et al. New entomological and virological data on the vectors of sylvatic yellow fever in Brazil. J Braz Assoc Advanc Sci 1992:44:136-42.
4. Vasconcelos PFC, Rodrigues SG, Dégallier N, Moraes MAP, Travassos da Rosa JFS, Travassos da Rosa ES, et al. An epidemic of sylvatic yellow fever in the southeast region of Maranhão State, Brazil, 1993-1994: Epidemiologic and entomological findings. Am J Trop Med Hyg, 1997:57:132-7.
5. Nobre A, Antezama D, Tauil PL. Febre amarela e dengue no Brasil: epidemiologia e controle. Rev Soc Bras Med Trop 1994:27(Suppl III):59-66.
6. World Health Organization. Prevention and control of yellow fever in Africa. Geneva: The Organization; 1985.
7. Monath TP. Yellow fever: a medically neglected disease. Report on a seminar. Rev Infect Dis 1987:9:165-75.
8. Monath TP. Yellow fever. In: Monath TP, editor. The arboviruses: epidemiology and ecology. Boca Raton (FL): CRC Press; 1988. p. 139-231.
9. Robertson SE, Hull BP, Tomori O, Bele O, LeDuc JW, Esteves K. Yellow fever. A decade of reemergence. JAMA 1996:276:1157-62.
10. Pan American Health Organization. Casos y muertes por fiebre amarilla en región de las Américas. Washington: The Organization; 1999.
11. Mondet B, Travassos da Rosa APA, Vasconcelos PFC. Les risques d'épidémisation urbaine de la fièvre jaune au Brésil pour le vecteurs *Aedes aegypti* et *Aedes albopictus*. Bull Soc Pathol Exot 1996:89:107-14.
12. Walter SD, Hildreth SW, Beaty BJ. Estimation of infection rates in population of organisms using pools of variable size. Am J Epidemiol 1980:112:124-8.
13. Shope RE. The use of a microhemagglutination-inhibition test to follow antibody response after arthropod-borne virus infection in a community of forest animals. Anais Microbiol (Rio de Janeiro) 1963:11(parte A):167-71.
14. Kuno G, Gomez I, Gubler DJ. Detecting artificial antidengue IgM immune complexes using an enzymelinked immunosorbent assay. Am J Trop Med Hyg 1987:36:153-9.
15. Beaty BJ, Calisher CH, Shope RE. Arboviruses. In: Schmidt NJ, Emmons E, editors. Diagnostic procedures for viral, rickettsial and chlamydial infections. 6th ed. Washington: American Public Health Association; 1989. p. 797-855.
16. Hall WC, Crowell TP, Watts DM, Barros VLRS, Kruger H, Pinheiro FP, et al. Demonstration of yellow fever and dengue antigens in formalin-fixed paraffin-embedded human liver by immunohistochemical analysis. Am J Trop Med Hyg 1991:45:408-17.
17. Monath TP. Yellow fever and dengue–the interactions of virus, vector and host in the re-emergence of epidemic disease. Sem Virol 1994:65:133-45.
18. Wang E, Weaver AC, Shope RE, Tesh R, Watts DM, Barrett ADT. Genetic variation in yellow fever virus: duplication in the 3' noncoding region of strains from Africa. Virology 1996:225:274-81.
19. Soper FL. Jungle yellow fever: new epidemiological entity in South America. Rev Hyg Saúde Públ 1936:10:107-44.
20. Strode GK, editor. Yellow fever. New York: McGraw-Hill; 1951.
21. Dégallier N, Travassos da Rosa APA, Vasconcelos PFC, Guerreiro SC, Travassos da Rosa JFS, Hervé JP. Estimation du taux de survie, de la densité relative et du taux d'infection d'une population d'*Haemagogus janthinomys* Dyar (Diptera, Culicidae) ayant fourni des souches de fièvre jaune en Amazonie Brésilienne. Bull Soc Pathol Exot 1991:84:386-97.
22. Gubler DJ. *Aedes aegypti* and *Aedes aegypti*-borne disease control in the 1990s: top down or bottom up. Am J Trop Med Hyg 1989:40:571-8.
23. Vasconcelos PFC, Travassos da Rosa APA, Dégallier N, Travassos da Rosa JFS, Pinheiro FP. Clinical and ecoepidemiological situation of human arboviruses in Brazilian Amazonia. Braz J Assoc Advanc Sci 1992:44:117-24.
24. Dégallier N, Travassos da Rosa APA, Vasconcelos PFC, Figueiredo LTM, Travassos da Rosa JFS, Rodrigues SG, et al. La dengue et ses vecteurs au Brésil. Bull Soc Pathol Exot 1996:89:128-36.
25. Van der Stuyft P, Gianella A, Pirard M, Cespedes J, Lora J, Peredo C, et al. Urbanisation of yellow fever in Santa Cruz, Bolivia. Lancet 1999:353:1558-62.
26. Monath TP, Nasidi A. Should yellow fever vaccine be included in the expanded program of immunization in Africa? A cost-effectiveness analysis for Nigeria. Am J Trop Med Hyg 1993:48:274-99.
27. Vasconcelos PFC, Travassos da Rosa APA, Pinheiro FP, Rodrigues SG, Travassos da Rosa ES, Cruz ACR, et al. *Aedes aegypti*, dengue and re-urbanization of yellow fever in Brazil and other South American countries-past and present situation and future perspectives. WHO Dengue Bulletin (New Delhi) 1999;23:55-66.
28. Groot H, Bahia-Ribeiro R. Neutralizing and haemagglutination-inhibiting antibodies to yellow fever 17 years after vaccination with 17D vaccine. Bull World Health Organ 1962:27:699-707.
29. Poland JD, Calisher CH, Monath TP, Downs WG, Murphy K. Persistence of neutralizing antibody 30-35 years after immunization with 17D yellow fever vaccine. Bull World Health Organ 1981:59:895-900.
